# Plasma Protein Carbonyls as Biomarkers of Oxidative Stress in Chronic Kidney Disease, Dialysis, and Transplantation

**DOI:** 10.1155/2020/2975256

**Published:** 2020-11-24

**Authors:** Graziano Colombo, Francesco Reggiani, Claudio Angelini, Silvia Finazzi, Emanuela Astori, Maria L. Garavaglia, Lucia Landoni, Nicola M. Portinaro, Daniela Giustarini, Ranieri Rossi, Annalisa Santucci, Aldo Milzani, Salvatore Badalamenti, Isabella Dalle-Donne

**Affiliations:** ^1^Department of Biosciences (Department of Excellence 2018-2022), Università degli Studi di Milano, Milan I-20133, Italy; ^2^Humanitas Clinical and Research Center-Nephrology Unit, Rozzano I-20089, Italy; ^3^Dipartimento di Biotecnologie Mediche e Medicina Traslazionale, Università degli Studi di Milano, Rozzano I-20089, Italy; ^4^Department of Biotechnology, Chemistry and Pharmacy (Department of Excellence 2018-2022), University of Siena, Siena I-53100, Italy

## Abstract

Accumulating evidence indicates that oxidative stress plays a role in the pathophysiology of chronic kidney disease (CKD) and its progression; during renal replacement therapy, oxidative stress-derived oxidative damage also contributes to the development of CKD systemic complications, such as cardiovascular disease, hypertension, atherosclerosis, inflammation, anaemia, and impaired host defence. The main mechanism underlying these events is the retention of uremic toxins, which act as a substrate for oxidative processes and elicit the activation of inflammatory pathways targeting endothelial and immune cells. Due to the growing worldwide spread of CKD, there is an overwhelming need to find oxidative damage biomarkers that are easy to measure in biological fluids of subjects with CKD and patients undergoing renal replacement therapy (haemodialysis, peritoneal dialysis, and kidney transplantation), in order to overcome limitations of invasive monitoring of CKD progression. Several studies investigated biomarkers of protein oxidative damage in CKD, including plasma protein carbonyls (PCO), the most frequently used biomarker of protein damage. This review provides an up-to-date overview on advances concerning the correlation between plasma protein carbonylation in CKD progression (from stage 1 to stage 5) and the possibility that haemodialysis, peritoneal dialysis, and kidney transplantation improve plasma PCO levels. Despite the fact that the role of plasma PCO in CKD is often underestimated in clinical practice, emerging evidence highlights that plasma PCO can serve as good biomarkers of oxidative stress in CKD and substitutive therapies. Whether plasma PCO levels merely serve as biomarkers of CKD-related oxidative stress or whether they are associated with the pathogenesis of CKD complications deserves further evaluation.

## 1. Introduction

**Figure 1:**
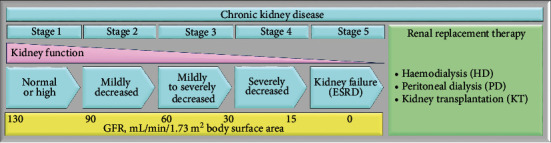
Five-stage classification system for CKD. During the progression of CKD, the decrease in kidney function, evaluated by the glomerular filtration rate (GFR), leads to a variety of disturbances in body homeostasis. The accumulation of uremic toxins, the increase in signs of volume overload, the worsening of hypertension, and the induction of metabolic and hormonal disturbances are typical of CKD patients. The progression of CKD often leads to a decline in residual renal function (RRF), eventually leading to renal replacement therapy (i.e., haemodialysis, peritoneal dialysis, and kidney transplantation).

Chronic kidney disease (CKD) has a worldwide prevalence of around 8-16%, and it is declared by the World Health Organization (WHO) as an ever-increasing public health problem [1]. CKD is usually characterized by albuminuria and/or decreased glomerular filtration rate (GFR), which is the volume of plasma filtered by the glomeruli per unit of time. A five-stage classification system for CKD has been established by the Kidney Disease Improving Global Outcomes (KDIGO) (Figure 1) [2]. Patients with stage 1-3 CKD are frequently asymptomatic. In CKD stages 1 and 2, GFR may be normal or borderline normal; hence, reduced GFR alone does not clinch the diagnosis. Other elements due to tubular disorders, such as albuminuria, the presence of a pathological urine sediment, electrolyte unbalance, or histologic and structural abnormalities detected by imaging, can be useful to establish the diagnosis of CKD stages 1 and 2 [2]. Clinical manifestations from low kidney function typically appear in stages 4 and 5. Patients with CKD show a progressive decline in kidney function, and they develop end-stage renal disease (ESRD, i.e., CKD stage 5), where renal replacement therapy (RRT) is needed to ensure ESRD patient survival. RRT is achieved by haemodialysis (HD), peritoneal dialysis (PD), and/or kidney transplantation (KT). Compared to the general population, CKD patients have a higher risk of premature death, primarily because of cardiovascular diseases (CVDs) [3, 4]. Traditional risk factors (such as age, diabetes, left ventricular hypertrophy, dyslipidemia, and hypertension) are predictive of CVD mortality in CKD patients [5]. In addition, CVD can also arise from nontraditional risk factors, including inflammation and oxidative stress, which are highly prevalent in CKD patients [4, 6–9].

Inflammation and oxidative stress interplay in a self-perpetuating vicious circle and drive CKD progression, CVD, and other numerous complications such as malnutrition, atherosclerosis, coronary artery calcification, heart failure, anaemia, and mineral and bone disorders [10–13]. In fact, patients with CKD typically suffer from chronic inflammation and have severely impaired antioxidant systems, which worsen gradually with the progression of renal failure [10]. Inflammation is characterized by an increase in inflammatory markers, including cytokines (such as interleukin-6, interleukin-1, tumour necrosis factor-*α*, and adipokines), acute phase proteins (mainly C-reactive protein), and adhesion molecules, which are associated with many complications during CKD, as clinical studies have demonstrated [11]. Many factors contribute to chronic inflammation in CKD, including the increased production of proinflammatory cytokines, oxidative stress, acidosis, chronic and recurrent infections, intestinal dysbiosis, and altered adipose tissue metabolism [12]. Inflammation contributes to the progression of CKD, oxidative stress, insulin resistance, endothelial dysfunction, mineral and bone disease, anaemia, and resistance to erythropoietin [11]. High levels of oxidative stress have been found in the early stages of CKD, which increase in parallel with the progression to ESRD [13] and even more in patients undergoing HD [14, 15]. In particular, HD induces inflammation and oxidative stress due to loss of antioxidants during the dialysis session and activation of white blood cells, which generate ROS [16]. Compared to HD, PD is a more biocompatible dialysis modality that induces a lower level of oxidative stress, mainly due to the composition of PD solutions (low pH, high lactate content, increased osmolarity, high glucose concentration, and related degradation products) [17, 18]. In particular, HD induces inflammation and oxidative stress due to loss of antioxidants during the dialysis session and activation of white blood cells, which generate ROS [19]. Compared to HD, PD is a more biocompatible dialysis modality that induces a lower level of oxidative stress, mainly due to the composition of PD solutions (low pH, high lactate content, increased osmolarity, high glucose concentration and related degradation products) [20, 21].

Oxidative stress has also been associated with the production of highly reactive intermediates during inflammation; ROS, for their part, further enhance the inflammatory response by triggering proinflammatory mediators. In the kidneys, ROS are mainly produced by the mitochondrial respiratory chain and by the different isoforms of the enzyme NADPH oxidase. Oxidative stress is responsible for progressive renal damage, which can lead to renal ischemia, lesions to the glomeruli, cell death, and apoptosis, exacerbating the severe inflammatory processes. Further, oxidative stress is also responsible for several risk factors for CKD, such as diabetes, hypertension, and atherosclerosis [11]. Several biomarkers of oxidative stress, such as malondialdehyde, oxidized low-density lipoprotein, advanced glycation end products, and 7,8-dihydro-8-oxo-2′-deoxyguanosine, have increased levels in patients with CKD [11]. However, their specificity as a biomarker of oxidative stress can be questionable, as in the case of oxidized low-density lipoprotein, which is most commonly measured in plasma or isolated lipoprotein by immunological methods using one of three different antibodies, each of which has methodological limitations [22]. All methods available for the detection of malondialdehyde show pitfalls, including the numerous commercial kits that lack specificity, making their significance for clinical practice dubious [22]. The thiobarbituric acid reactive substance assay to detect malondialdehyde reveals reproducibility and reliability when combined with HPLC, although it requires individual sample processing and its validity as a biomarker of *in vivo* oxidative stress remains uncertain, making it less suitable for routine clinical use [22].

CKD is also characterized by the accumulation of uremic toxins released from the intestinal tract, which have become clinically relevant in CKD progression and are tightly related to many CKD-associated systemic complications, including inflammation, oxidative stress, and decreased production of nitric oxide by endothelial cells [23]. The proinflammatory state, the enhanced oxidative stress, and the accumulation of uremic toxins also cause endothelial damage. Under uremia, endothelial cells produce danger-associated molecular patterns (molecules released by stressed, damaged, or necrotic cells that act as endogenous danger signals to promote and perpetuate a noninfectious inflammatory response), which induce the expression of adhesion molecules, the production of proinflammatory cytokines, and an enhanced production of ROS in endothelial cells [24]. Uremic toxins are involved in the inflammatory state in CKD and contribute to many uremia-associated dysfunctions [11]. In fact, several studies have shown that uremic toxins increase the levels of TNF-*α* and IL-6 and cause an exacerbation of the inflammatory state through the increase in ROS production [11].

**Figure 2:**
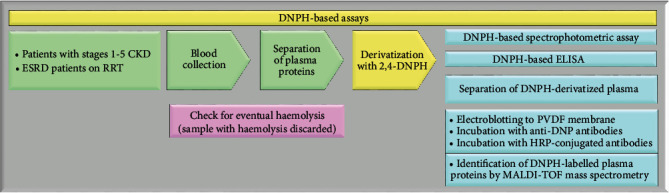
DNPH-based assays for PCO detection. Assays for the detection of PCO involve the derivatization with 2,4-DNPH, leading to the formation of a stable dinitrophenylhydrazone product. PVDF: polyvinylidene fluoride membrane; HRP: horseradish peroxidase; MALDI-TOF: matrix-assisted laser desorption/ionization time-of-flight; RRT: renal replacement therapy.

Protein carbonyls (PCO) are among the most successful biomarkers of oxidative stress and are associated with disease state and treatment in multiple illnesses [22, 25–28]. The easy sampling of plasma proteins and the relatively long half-life of many of them make plasma PCO an attractive biomarker of oxidative stress in CKD [29, 30]. The most commonly used methods for quantifying PCO rely on derivatization with 2,4-dinitrophenylhydrazine (DNPH), which specifically reacts with PCO associated with aldehydes and ketones but does not react with other carbonyl-containing functional groups such as carboxylic acids and esters. DNPH generates the stable 2,4-dinitrophenylhydrazone (DNP) adduct (Figure 2) that absorbs UV light; therefore, PCO can be detected by a spectrophotometric assay [31]. DNPH-derivatized PCO can also be detected by specific anti-DNP antibodies by enzyme-linked immunosorbent assay (ELISA) and Western blot. The ELISA makes use of biotin-linked anti-DNP antibodies that bind DNP-derivatized proteins and allow detection with streptavidin-HRP [32, 33]. Carbonylation of specific plasma proteins is often detected by Western blot. After derivatization with DNPH, plasma proteins are separated by one-dimensional sodium dodecyl sulphate polyacrylamide gel electrophoresis (1D SDS-PAGE) or by two-dimensional gel electrophoresis (2D-GE), electrotransferred from the gel to a polyvinylidene fluoride (PVDF) membrane, and then, PCO can be immunodetected using primary anti-DNP antibodies and horseradish peroxidase- (HRP-) conjugated secondary antibodies [34, 35]. For mass spectrometry (MS) analysis, protein bands (or spots) can be excised from the gel, in-gel reduced, thiol-alkylated, digested with trypsin, and identified by matrix-assisted laser desorption/ionization time-of-flight (MALDI-TOF)/MS mass fingerprinting [36–38]. PCO can also be detected by fluoresceinamine, a molecule that, unlike DNPH, labels PCO derived from metal-catalysed oxidative modification. Climent and colleagues demonstrated that fluoresceinamine labels specifically the *γ*-glutamyl semialdehyde group [39].

In this review, we summarize and discuss the main studies that have assessed plasma PCO levels in CKD, dialysis, and kidney transplantation and the potential role of protein carbonylation in driving CKD progression.

## 2. Plasma Protein Carbonyls in CKD

Although a high prevalence of oxidative stress in CKD is now well-established [4, 6, 30], few studies measured biomarkers of oxidative stress in people with CKD. Increased oxidative stress in patients with CKD stage 3 or higher, including ESRD, is demonstrated by an increase in plasma protein thiol oxidation, PCO, advanced oxidation protein products (AOPPs), and protein-bound di-tyrosines ([40–42] and citations therein). However, the properties of the oxidative modifications, e.g., the transience of cysteine modifications, their low abundance, e.g., protein-bound di-tyrosines, or methodological issues concerning the reproducibility, accuracy, and reliability of their detection, e.g., AOPPs, limit their applicability in clinical practice. Considering that PCO are chemically stable, their concentration is often higher than that of other biomarkers (since PCO formation can derive from different mechanisms), and validated detection methods are available [22, 34, 43], the plasma PCO assay has some advantages over other methods to assess oxidative stress and protein oxidative damage in clinical practice. In particular, the methods that seem to be most applicable in clinical settings are ELISA, as commercial kits are available, and HPLC, both of which allow for the rapid processing of many samples, the use of internal/external standards, and comparison of samples under constant conditions [22].

Eight studies examined the plasma PCO level in patients with CKD at various stages by spectrophotometric assay and ELISA (Table 1). Carbonylation of individual plasma proteins was measured by Western blot only in one study [44]. Four out of eight studies examined plasma PCO levels in patients with CKD compared with healthy subjects [45–47], while the other four examined plasma PCO levels in CKD patients at various stages [48–51].

Oberg et al. [45] compared 60 adult/elderly patients with CKD stages 3-5 (67 ± 14 years, 29 of whom with diabetes mellitus) and healthy control subjects (51.4 ± 1.7 years) (Table 1), showing that plasma PCO levels were significantly higher in patients with CKD than in healthy control subjects, as subsequently confirmed by other studies [46, 48]. As no significant correlation between GFR and plasma PCO content was observed [45], the authors suggested that PCO can undergo renal clearance primarily via renal tubular metabolism rather than glomerular filtration; plasma PCO content could therefore be largely regulated by proximal tubular function [45].

The plasma PCO level increased in parallel with decreased renal function (measured as creatinine clearance) (*R* = −0.692, *p* < 0.0001) in elderly patients (60.9 ± 15.2 years) with CKD at stages 1-5 [48]; in addition, a significantly positive correlation was observed between plasma PCO and blood urea nitrogen (BUN) (*R* = 0.695, *p* < 0.0001) [48]. Evaluation of plasma protein carbonylation in elderly patients with CKD at stages 1-4 showed that the PCO level in patients classified at stage 4 was higher than that of patients at stages 3, 2, and 1 (*p* < 0.001) [50]. A further study conducted in elderly patients with CKD stages 2-5 highlighted that plasma levels of PCO in CKD stage 5 were significantly higher than in stage 2 (*p* = 0.003), stage 3 (*p* = 0.015), and stage 4 (*p* = 0.011) [49]. On the contrary, the plasma PCO level in young patients (aged from 1.4 to 18.6 years) with CKD stages 1-5 did not depend on the degree of renal failure [51], probably as a consequence of the different diseases underlying kidney dysfunction in young people compared to the elderly. In fact, CKD is more commonly caused by diabetes mellitus and long-lasting hypertension in the elderly, while it is prevalently due to congenital abnormalities of the kidneys and urinary tract in young patients [51]. One study found significantly higher plasma PCO levels in CKD patients, both on conservative therapy (CT) and HD, than in healthy control adults [47]. This study also showed a negative correlation between plasma PCO levels and creatinine clearance [47] that was confirmed [49] or not [45] by other studies.

The plasma PCO level has been shown to have a negative correlation with GFR (*R* = −0.26, *p* < 0.05) and a positive correlation with C-reactive protein (CRP) (*R* = 0.49, *p* < 0.0001) and fibrinogen (*R* = 0.30, *p* < 0.01) levels [49]. Assessment of carbonylation of plasma albumin in elderly patients with CKD stages 2-5 and healthy control subjects by 1D Western blot analysis showed increasing carbonylation of albumin in parallel with the severity of CKD, which reached statistical significance at CKD stages 3 and 4 (*p* < 0.01, compared to healthy control subjects) [44].

## 3. Plasma Protein Carbonyls in Haemodialysis (HD)

Patients receiving HD, the most common type of dialysis, show a high prevalence of inflammation and oxidative stress [11, 13, 14, 52] and are exposed to additional health risk factors determined by the procedure itself (e.g., rapid changes in plasma electrolyte levels, haemodynamic stresses because of intra- and interdialytic changes in cardiac filling, and fluctuations of blood pressure). Inflammatory response can be caused by the use of synthetic membranes during HD [53] as well as by dialysate impurities, as biomarkers of inflammation and oxidative stress appear significantly lower in patients treated with ultrapure versus standard dialysate [54]. Various mechanisms have been proposed to account for the additional oxidative stress observed in patients following HD, including the activation of neutrophil NADPH oxidase, which provokes inflammation with the release of reactive oxygen species (ROS) [14, 55], and the depletion of circulating low-molecular-weight dialyzable antioxidants [56]. Some typical complications in patients undergoing HD can further exacerbate oxidative stress. For example, anaemia is a frequent and early complication of CKD and the treatment with iron can increase oxidative stress levels. Anaemia prevalence increases with worsening of renal function, involving over 50% of patients at stage 4 and virtually almost 100% of patients receiving HD [57]. Erythropoiesis is limited by the low iron availability [58], deriving from either absolute or functional deficiency and from the iron block due to underlying inflammatory status [59]. Iron deficiency is common in patients with ESRD on HD [60] overall; they lose on average 1-2 g of iron per year, and some of them as much as 4 to 5 g per year [61]. In the absence of concomitant iron supplementation, erythropoietin therapy does not affect oxidative stress [62]. Nevertheless, intravenous iron supplementation is one of the most used interventions in patients with CKD to correct anaemia [63], even if it further aggravates oxidative stress [64]. Recently, iron overload has been shown to increase plasma PCO levels in ESRD patients on HD [65]. Moreover, plasma PCO were positively associated with ferritin level (*R* = 0.35, *p* = 0.01) [65].

Biomarkers of inflammation are elevated in ESRD patients on HD [66–69]. The level of CRP increases in 30-60% of patients receiving HD, and it is closely associated with the progression of atherosclerosis, cardiovascular morbidity, and mortality [70]. Biomarkers of oxidative stress, such as *S*-thiolated proteins [71–73] and protein-bound di-tyrosines [40, 74], are also heightened in these patients. Additionally, 24 studies measured plasma PCO levels in ESRD patients on HD (Table 2), 18 of which determined that plasma PCO levels in haemodialysed patients are higher than in healthy subjects (Table 2).

Colombo et al. [74] were the first to point out significant differences in plasma PCO levels between healthy subjects and ESRD patients on HD. Many other studies later confirmed those findings (Table 2). Interestingly, Caimi et al. [47] also showed that patients receiving HD had higher plasma PCO levels not only when compared with healthy control subjects but also in comparison with CKD patients on CT.

A prospective cohort study (12-month period) showed that, at baseline, plasma PCO levels were significantly higher in patients with ESRD before starting the HD therapy than in healthy subjects [75]. After the initiation of HD, there were no significant changes both in plasma PCO content and in plasma concentration of inflammatory biomarkers, which remained stable over a 12-month period [75]. Other studies conducted in ESRD patients on HD reported an increase in IL-6 levels during a 3-year follow-up period [76] and a positive correlation between plasma PCO levels (higher than in healthy subjects) and the duration of HD (3 to 120 months) (*R* = 0.364, *p* < 0.01) [77]. Differently, another study showed that plasma PCO content was not significantly different in ESRD patients receiving HD for up to eleven years (Table 2) [78].

A study evaluated the within- and between-individual variability of plasma PCO levels in ESRD patients on HD, with PCO measurements every two weeks for ten weeks (six measures) [79]. Within-individual coefficients of variation (CVs) and between-individual CVs for PCO were, respectively, 16.3% (range 8.4–29.5%) and 19.5% (range 15.6–24.5%). PCO variability was not affected by various personal and external factors such as dietary antioxidant intake, medications, and clinical and demographic parameters. However, the higher number of males (10 men and 4 women) participating in the study may have influenced the ability to look at the effect of sex [79].

Some studies examined plasma PCO levels before (pre-HD) and after (post-HD) a single HD session (Table 2). Among these, Ward et al. [80] were the first to measure plasma PCO levels pre-HD and post-HD. They reported that plasma PCO levels increased slightly, but significantly over the course of dialysis [80]. Several other studies found that the post-HD plasma PCO content was higher than the pre-HD one [41, 47, 81–84]. Caimi et al. [84] detected increased levels of PCO in CKD patients on HD in comparison with normal controls before and, especially, after the HD session, but they did not find any difference in PCO after subdividing haemodialysed patients according to their dialysis vintage (i.e., length of time on dialysis) or the type of filter employed for HD. Interestingly, Colombo et al. [41] divided ESRD patients on HD into two groups according to sex, and they reported that pre-HD and post-HD plasma PCO levels in females were significantly different while in males were not. This finding suggests that female ESRD patients may be more susceptible to oxidative stress induced by the HD session than male ESRD patients [41]. As a consequence, the female sex could be considered a “risk factor” associated with HD-induced plasma protein carbonylation in ESRD patients. Two studies, on the other hand, reported that plasma PCO concentration did not change during the HD session [85] and that, in anuric haemodialysed patients with or without cardiovascular diseases, post-HD plasma PCO levels decreased [86]. However, it is not specified whether these differences are statistically significant [86].

In ESRD patients undergoing HD or PD, serum albumin is considered a biomarker of nutritional status and inflammation and a predictor of mortality [87, 88]. Some studies have shown that albumin is the major carbonylated protein in ESRD patients on HD (Table 2). In 2001, Himmelfarb and McMonagle [89] reported, for the first time, that the carbonylation of albumin accounts for almost all the excess plasma protein oxidation observed in haemodialysed patients when compared to healthy subjects. Later studies confirmed and reinforced this finding. The carbonyl content of purified albumin, detected by spectrophotometric analysis and immunoblot, was much higher in ESRD patients on HD than in healthy subjects [90]. Carbonylation of albumin increased in correlation with CKD stage severity, attaining significance at stages 3 and 4 (*p* < 0.01, compared to healthy controls), and reached even higher levels in patients undergoing HD [44]. As mentioned above, in haemodialysed patients, intravenous iron administration substantially increases carbonylation of plasma proteins [65], including albumin carbonylation [91]. Comparing healthy control subjects, ESRD patients undergoing HD without intravenous iron administration, and ESRD patients undergoing HD with intravenous iron administration, only albumin was found to be significantly carbonylated in haemodialysed patients, and intravenous iron administration increased the albumin carbonylation [91].

Advanced age (>65 years) has been associated with hypoalbuminemia (serum albumin level < 3.8 g/dL) in two large cross-sectional studies [92, 93]. Hypoalbuminemia in ESRD patients on HD is primarily associated with systemic inflammation [94] and confers a greater mortality risk [95, 96]. Danielski et al. [97] reported that both plasma PCO levels and albumin carbonyl content were significantly increased in normoalbuminemic and hypoalbuminemic ESRD patients on HD in comparison to healthy subjects, even if the difference between hypoalbuminemic and normoalbuminemic haemodialysed patients did not reach the statistical significance [97].

Albumin is not the only protein whose carbonylation increases in ESRD patients on HD [37]. Using Western blot with anti-DNP antibodies and MALDI-TOF/MS mass fingerprinting associated with nano-LC-MS/MS analysis, Pavone et al. [37] showed that post-HD plasma PCO levels were significantly increased compared to pre-HD levels and that carbonylation targets numerous plasma proteins. The same authors used MALDI-TOF/MS mass fingerprinting to identify carbonylated proteins in blood samples before and after the HD session carried out with ethylene vinyl alcohol and cellulose diacetate membranes. *α*2-Macroglobulin, chain A *α*1-antitrypsin, fibrinogen *γ* chain, immunoglobulin *γ* 1, proapolipoprotein, transferrin, and albumin were found as the main carbonylated plasma proteins after HD [36].

## 4. Plasma Protein Carbonyls in Peritoneal Dialysis (PD)

PD is an alternative to HD and is used by approximately 200,000 ESRD patients worldwide, representing approximately 7% of the total dialysis population [98]. In PD, the peritoneal membrane acts as a dialyzing membrane. To achieve this, a dialysis solution (dialysate) is instilled in the peritoneal cavity through a peritoneal catheter. After a dwell time, the dialysate is drained out. How long the dialysate is present in the peritoneal cavity, how many times the dialysate is changed, and the duration of the dwell time depend on individual patient requirements. While the dialysate is present in the peritoneal cavity, across the peritoneal membrane there is a transport of solutes and water between the blood in the peritoneal capillaries and the dialysis solution, which is typically rendered hyperosmolar through the addition of glucose or other osmotic agents. Through this mechanism, the elimination of waste products and the correction of fluid and electrolyte imbalances are achieved [99]. Even though HD and PD can be viewed as equivalent therapies and used as primary therapy for ESRD patients [100], there are important differences between them. PD exposes patients daily to greater amounts of glucose loading, leading to a much higher prevalence of insulin resistance, dyslipidemia, and metabolic syndrome [101]. PD may also accelerate the development of atherosclerosis lesions through increased lipid oxidation and glycosylation [102]. Otherwise, patients with ESRD undergoing HD are exposed to a greater risk of CVDs because they show a more rapid decline of residual renal function (RRF) [103] and a more hyperdynamic status due to the arteriovenous fistula and the extracorporeal circulation [104]. Although PD is considered a less invasive therapy than HD, it produces chronic inflammation in the peritoneal cavity leading to an increased level of proinflammatory cytokines, which alters peritoneal membrane integrity [105]. Several lines of evidence indicated that oxidative metabolism in peripheral and peritoneal phagocytes is activated during PD with conventional dialysate, which is characterized by a high concentration of glucose, glucose degradation products, low pH, and high osmolality [106]. Bioincompatibility of PD solutions also seems to play a central role in the oxidative stress increase [107].

Five studies compared plasma PCO levels in patients with ESRD undergoing PD or HD, while one study examined plasma PCO content in ESRD patients on PD and healthy individuals (Table 3). Erdoğan et al. [108] compared plasma PCO content of patients with ESRD undergoing HD or PD with that of healthy individuals, showing that plasma PCO levels in ESRD patients on HD or PD are similar to those of healthy controls. Otherwise, another study showed that plasma PCO levels in ESRD patients on PD were lower than in haemodialysed patients, maybe because HD is associated with higher protein oxidation or because patients undergoing PD had greater RRF [109]. Conversely, two more recent studies showed that plasma PCO levels were higher in ESRD patients undergoing PD than in those undergoing HD [50, 110], although in one of the two studies it is not clear whether differences were statistically significant [50]. Another investigation proved that, in ESRD patients on PD, there was a highly significant positive correlation between copper/zinc ratio, the levels of CRP, and plasma PCO levels [111], whereas copper/zinc ratio was negatively correlated with the percentages of B- and T-lymphocytes and the ratio of CD4/CD8 antigens. Therefore, the authors suggested that, in ESRD patients on PD, elevated copper/zinc ratios are associated with increased oxidative stress and inflammation [111].

Mitrogianni et al. [44] estimated the carbonylation of plasma albumin in ESRD patients undergoing HD or PD compared to healthy control subjects by Western blot, showing that albumin carbonylation was higher in ESRD patients on HD, while it did not differ in ESRD patients on PD compared to controls. They suggested that PD may be more biocompatible, avoiding the generation of excess oxidative burden. Lack of contact of the blood with the dialysis membranes and less usage of intravenous iron administration might explain, at least in part, the low levels of plasma PCO observed in ESRD patients undergoing PD. The quite important albumin losses in ESRD patients on PD, replaced by newly synthesized albumin, may contribute to the lower albumin carbonylation [44].

## 5. Plasma Protein Carbonyls in Kidney Transplantation (KT)

KT is considered the best therapeutic option in ESRD, because it permits a higher quality of life compared to HD and PD. Moreover, KT presents the lowest mortality rates, around 1.5-7% per year [112]. In KT anaemia, in addition to hyperhomocysteinemia, it can induce oxidative stress [113]. Oxidative stress and inflammation can produce graft tissue damages because of fibrosis and nephron losses by necrosis or apoptosis [114].

Two studies examined plasma PCO levels before and after KT. A prospective cohort study evaluated time-dependent changes in biomarkers of inflammation and oxidative stress (plasma PCO levels) in 19 patients (mean age 38.3 ± 13.7 years, 11 men and 8 women), comparing them to 50 healthy control subjects (mean age 48.2 ± 16 years, 18 men and 32 women) [115]. This study reported that patients had substantial improvements in inflammatory biomarkers and plasma PCO levels after the restoration of kidney function by transplantation. Plasma PCO levels decreased rapidly, with significant changes notable within the first postoperative week (*p* < 0.001); final posttransplant levels of plasma PCO in recipients were not statistically different from those of healthy subjects (*p* < 0.05). This study also showed that CRP levels decreased significantly from baseline within two months after renal transplantation (*p* < 0.001) [115]. The second study investigated plasma protein carbonylation in 21 patients (mean age 36 ± 17 years, men 50%) who underwent a living donor KT and were evaluated before the transplantation and analyzed longitudinally after a mean follow-up time of nine months [35]. This study showed that plasma PCO levels declined from seven to 11 months after KT. Plasma PCO content was significantly reduced after KT (1.4 ± 0.4 nmol/mg albumin) compared to pretransplantation (2.0 ± 1.4 nmol/mg albumin, *p* < 0.05). The study also revealed a significant correlation between CRP and plasma PCO levels after the transplantation (*R* = 0.65, *p* < 0.005) [49].

## 6. Conclusion and Perspectives

Plasma PCO levels are quite heterogeneous both in CKD patients (Table 1), in patients on RRT (Tables 2 and 3), and in healthy control individuals (Tables 1–3). A cause of PCO variability could be the use of different methods to measure plasma PCO levels due to the lack of a reference method. Nevertheless, even when the same methodology was used, a critical emerging aspect is the high variability of measurements (Table 1). The absolute value of plasma PCO content measured by ELISA in control and CKD subjects seems to spread over two orders of magnitude (e.g., 0.029 and 0.061 nmol/mg protein [45], 0.440 and 0.709 nmol/mg protein [47], and 3.63 and 7.41 nmol/mg protein [46]). This variability needs to be reduced by standardizing references or procedures to make comparable data obtained at different times and laboratories. The aim is to give solidity as well as diagnostic and prognostic value to PCO, an established biomarker of oxidative stress.

A problem with the DNPH-based spectrophotometric assay may be that its results are frequently displayed in different units, e.g., nmol/mg protein, nmol/mg albumin, nmol/L, mmol/L, ng/*μ*L, and *μ*mol∗mL (Tables 1–3), making them particularly difficult to compare between different studies. In addition, a limit of the DNPH-based spectrophotometric assay is that absorbance wavelengths of haemoglobin are similar to those of DNPH and this can interfere with DNPH measurement, leading to inaccurate estimation of plasma PCO levels [116]. Therefore, reproducible results can arise only from meticulous sample preparation (i.e., during plasma separation from red blood cells, haemolysis should be strictly avoided) (Figure 2).

RRF can further contribute to variability in plasma PCO levels among patients with ESRD undergoing dialysis. Moreover, in various studies, the inclusion/exclusion criteria of CKD patients are quite heterogeneous or even unspecified (Table 4—Supplementary Material). Moreover, age, sex, ethnicity, and lifestyle can also potentially result in PCO variability. Therefore, the preliminary results of these small studies should be confirmed in the future with a larger number of CKD patients and/or patients on RRT with homogeneous (or at least well-specified) inclusion/exclusion criteria and healthy control subjects with different demographic characteristics (e.g., age, sex, and ethnicity) and lifestyle (e.g., physical activity level and smoking status).

However, despite these problems, some findings emerge clearly from the studies conducted so far. Firstly, the results of the studies reported in Table 1, taken together, emphasized the fact that plasma PCO levels are increased in adult or elderly patients with CKD compared to healthy subjects. Even in the early stages of CKD, plasma PCO levels are elevated, and they increase from one stage to the next one, as the kidney function declines over time. This supports the conclusion that systemic oxidative stress appears already at the initial stages of CKD and it gradually increases along with the severity of the disease. Few data are present in the literature about PCO levels in people younger than 18 years. The only study in this setting showed that, in children and young patients with CKD stages 1-5, the concentration of plasma PCO did not depend on the stage of disease [51]. In addition, plasma PCO levels from adult or elderly patients with CKD seem to be correlated negatively with GFR [47–49] and positively with BUN [48], even if caution is necessary to interpret these small studies.

Secondly, studies measuring plasma PCO levels in ESRD patients on PD *vs.* ESRD patients on HD have generated conflicting results [44, 50, 108–110] (Table 3). Maybe this could be due to interfering factors such as the different RRF between ESRD patients undergoing HD or PD. In fact, RRF decreases more slowly in people undergoing PD than in those undergoing HD [117, 118], probably because of sudden drops in blood pressure typical of HD, where fluid is removed much more quickly during the short and frequent HD sessions as compared to the longer PD cycles. In addition, other factors can influence RRF decline, such as gender (particularly female gender as being associated with a stronger decline), nonwhite race (associated with a stronger decline), and comorbidities [103, 119]. A further limitation of these studies was the relatively small number of patients.

Thirdly, after the restoration of kidney function by transplantation, plasma PCO content lowers to levels similar to those of healthy control subjects. Although the populations involved were limited in size, several studies support the conclusion that KT reduces oxidative stress [49, 115, 120, 121].

In conclusion, the studies presented in this review demonstrate that oxidative stress is higher in CKD. Western blot analysis with anti-DNP antibodies showed that not all proteins in the plasma of CKD patients are prone to carbonylation, supporting the view that protein carbonylation in CKD is a selective rather than a random process. In patients with various stages of CKD [44] and in ESRD patients on HD, carbonylation affects albumin [41, 44, 89–91] and other proteins present in the plasma in lower amounts [36, 37]. Direct, or primary, carbonylation is a protein irreversible damage, an oxidative modification that cannot be reversed by antioxidant defences [22, 25, 122, 123]. The increased carbonylation of proteins directly leads to the central unsolved question: does the carbonylation of proteins have a direct pathological impact or is it a secondary phenomenon? Albumin, along with ascorbate and urate, represents the most important antioxidants in the plasma [124]. As albumin is carbonylated in CKD patients, including ESRD patients on HD, it can be hypothesized that, in these subjects, the plasma antioxidant defences are lower and, consequently, the risk for oxidative tissue damage is higher [125]. Several studies conducted in ESRD patients on HD have indeed demonstrated that albumin carbonylation can adversely affect its vasculoprotective capabilities [89, 90], fibrinogen carbonylation can contribute to the impaired clotting activity [126], and carbonylation of haptoglobin and ceruloplasmin [36, 37] can impair the antioxidant protective properties of these proteins.

Overall, all these studies point out that plasma protein carbonylation in CKD, and especially in ESRD patients undergoing HD, is not solely a secondary phenomenon. Despite the fact that the role of plasma PCO in CKD is often underestimated in clinical practice, emerging evidence continues to highlight that plasma PCO can serve as good biomarkers of oxidative stress in CKD and substitutive therapies, HD, PD, and KT. Whether plasma PCO levels merely serve as biomarkers of CKD- and RRT-related oxidative stress or whether they are associated with the pathogenesis of CKD complications deserves further evaluation. In this regard, it is interesting to note that advanced glycation end products (AGEs), i.e., glycated amino acid residues of proteins, contribute to the development of CKD [127]. AGEs are stable posttranslational modified proteins derived by the nonenzymatic reaction of reducing sugars and related metabolites with Arg and Lys residues, giving rise to indirect, or secondary, protein carbonylation. Proteolysis of AGEs produces glycated amino acids, or AGE-free adducts, which are cleared by the kidneys under healthy conditions but accumulate in plasma with the decline in GFR during CKD [127, 128]. AGEs also result from dicarbonyls derived from glucose degradation and absorbed from thermally processed dialysis fluids in RRT and from the so-called dicarbonyl stress, i.e., the accumulation of various dicarbonyl compounds that causes increased AGE formation in people with CKD [127, 129]. In patients with ESRD, plasma AGE-free adducts increased up to 18-fold on PD and up to 40-fold on HD, whereas the increase in AGE residues of plasma proteins was 2- to 5-fold [128]. Protein dysfunction and inactivation caused by AGE formation contribute to CKD development [127]. Indeed, several studies that investigated dysfunction of proteins modified by dicarbonyl compounds—the so-called dicarbonyl proteome—suggest that dicarbonyl stress is a key factor for the development of vascular renal inflammation, kidney and muscle fibrosis, which are critical to CKD progression and comorbidities, CVD, and muscle wasting [127, 130].

## Figures and Tables

**Table 1:** Plasma PCO levels in CKD stages 1-5.

Study year [Ref.]	CKD stages	CKD group (age and sex)	Control group (age and sex)	Analytical methods	PCO in CKD group	PCO in control group	*p* value
Oberg et al. 2004 [45]	3 to 5	(A) 60 patients (age 67 ± 14 years, 38 M and 22 F)	(H) 53 healthy subjects (age 51.4 ± 1.7 years, sex unspecified)	ELISA after derivatization with DNPH (commercial kit)	(A) Stages 3-5 0.061 nmol/mg protein (0.020–0.134) (a)	(H) 0.029 nmol/mg protein (0–0.154) (a)	(A) vs. (H) *p* < 0.001

Puchades Montesa et al. 2009 [46]	4	(A) 32 patients (age 65.29 ± 15.6 years, 26 M and 6 F)	(H) 67 healthy subjects (age 48.08 ± 19.11 years, 29 M and 38 F)	ELISA after derivatization with DNPH [139]	(A) Stage 4 7.41 ± 0.84 nmol/mg protein	(H) 3.63 nmol/mg protein (1.12) (b)	(A) vs. (H) *p* < 0.001

Mitrogianni et al. 2009 [44]	2 to 4	(A) 25 patients on stage 2 (age range 35–79 years, 15 M and 10 F) (B) 29 patients on stage 3 (age range 29–77 years, 18 F and 11 F) (C) 27 patients on stage 4 (age range 30–81 years, 14 M and 13 F)	(H) 20 healthy subjects (age range 29–78 years, 12 M and 8 F)	Western blot analysis after derivatization with DNPH (commercial kit)	(A) Stage 2 58.88 ± 3.87 a.u. (B) Stage 3 49.48 ± 2.80 a.u. (C) Stage 4 73.34 ± 6.00 a.u	(H_A) Healthy subjects 49.26 ± 4.02 a.u. (H_B) Healthy subjects 34.16 ± 3.94 a.u. (H_C) Healthy subjects 40.24 ± 6.34 a.u	(A) vs. (H_A) No significant variations (B) vs. (H_B) *p* < 0.05 (C) vs. (H_C) *p* < 0.05

Matsuyama et al. 2009 [48]	1 to 5	(A) 7 patients on stage 1-2 (age 51.3 ± 7.6 years, 5 M and 2 F) (B) 7 patients on stage 3a (age 58.2 ± 12.2 years, 4 M and 3 F) (C) 6 patients on stage 3b (age 71.3 ± 8.2 years, 5 M and 1 F) (D) 12 patients on stages 4-5 (age 63.3 ± 19.4 years, 8 M and 4 F)	—	Spectrophotometric assay after derivatization with DNPH (commercial kit)	(A) Stages 1-2 0.7 ± 0.1 nmol/mg protein (B) Stage 3a 0.8 ± 0.1 nmol/mg protein (C) Stage 3b 1.0 ± 0.2 nmol/mg protein (D) Stages 4-5 1.1 ± 0.2 nmol/mg protein	—	(A) vs. (D) *p* < 0.05

Aveles et al. 2010 [49]	2 to 5	68 patients (age 57 ± 12.6 years, 31 M and 37 F)	—	Spectrophotometric assay after derivatization with DNPH [136]	(A) Stage 2 0.8 ± 1.3 nmol/mg albumin (B) Stage 3 1.2 ± 0.9 nmol/mg albumin (C) Stage 4 1.0 ± 0.7 nmol/mg albumin (D) Stage 5 2.2 ± 1.6 nmol/mg albumin	—	(A) vs. (D) *p* = 0.003 (A) vs. (C) *p* = 0.015 (A) vs. (B) *p* = 0.011

Caimi et al. 2013 [47]	2 to 5	(A) 27 patients at stages 2-5 on conservative therapy (CT) (age 58.2 ± 7.6 years, 15 M and 12 F)	(H) 26 healthy subjects (age 43.54 ± 6.92 years, 17 M and 9 F)	ELISA after derivatization with DNPH (commercial kit)	(A) CT stages 2-5 0.709 ± 0.107 nmol/mg protein	(H) 0.440 ± 0.134 nmol/mg protein	(A) vs. (H) *p* < 0.001

Tbahriti et al. 2013[50]	1 to 4	(A) 28 patients on stage 1 (age 37 ± 13 years, 10 M and 18 F) (B) 28 patients on stage 2 (age 55 ± 11 years, 11 M and 17 F) (C) 28 patients on stage 3 (age 45 ± 15 years, 10 M and 18 F) (D) 18 patients on stage 4 (age 46 ± 14 years, 7 M and 11 F) (E) 40 HD patients (age 42 ± 11 years, 22 M and 18 F) (F) 25 PD patients (age 39 ± 15 years)	—	Spectrophotometric assay after derivatization with DNPH (commercial kit)	(A) Stage 1 0.56 ± 0.15 nmol/mg albumin (B) Stage 2 0.95 ± 0.13 nmol/mg albumin (C) Stage 3 1.04 ± 0.33 nmol/mg albumin (D) Stage 4 1.37 ± 0.36 nmol/mg albumin (E) HD patients 1.85 ± 0.16 nmol/mg albumin (F) PD patients 1.92 ± 0.13 nmol/mg albumin	—	(A-B-C-D) vs. (E) *p* < 0.001 (A-B-C-D) vs. (F) *p* < 0.001

Drożdż et al. 2016 [51]	1 to 4	(A) 11 patients on stages 1-2 (age 10.51 years; 5.04, 16.08) (B) 18 patients on stage 3 (age 11.33 years; 5.15, 16.33) (C) 14 patients on stage 4 (age 12.01 years; 8.70, 15.99) (D) 22 patients on stage 5 (age 11.61 years; 8.51, 15.20)	—	Spectrophotometric assay after derivatization with DNPH (commercial kit)	(A) Stage 1-2 1.15 nmol/mg protein (0.54, 1.32) (c) (B) Stage 3 1.24 nmol/mg protein (0.87, 1.69) (c) (C) Stage 4 1.64 nmol/mg protein (0.73, 2.41) (c) (D) Stage 5 1.23 nmol/mg protein (0.66, 2.05) (c)	—	No significant variations

Data are presented as the mean ± standard deviation (SD), in the reported studies, with the exception of (a) medians, with a range in parentheses, (b) median with interquartile range in parentheses, and (c) median with 25th–75th percentile. M: male; F: female; a.u.: arbitrary units; CKD: chronic kidney disease; PCO: protein carbonyls (carbonylated proteins); DNPH: 2,4-dinitrophenylhydrazine.

**Table 2:** Plasma PCO levels in HD.

Study year [Ref.]	HD patients (age and sex) and dialysis vintage	Healthy subjects (age and sex)	Analytical methods	Plasma PCO in HD patients	Plasma PCO in healthy subjects	*p* value
Himmelfarb at al. 2000 [131]	(A) 10 HD patients (sex and age unspecified) Dialysis vintage unspecified	(H) 10 healthy subjects (sex and age unspecified)	ELISA after derivatization with DNPH [32]	(A) 16.95 ± 2.62 *μ*mol/L	(H) 0.76 ± 0.51 *μ*mol/L	(A) vs. (H) *p* < 0.05

Himmelfarb and McMonagle 2001 [89]	(A) 25 HD patients (mean age 72.6 ± 2.0 years, 13 M and 12 F) Dialysis vintage unspecified	(H) 20 healthy subjects (age 62 ± 4 years, 17 M and 3 F)	ELISA after derivatization with DNPH (commercial kit) Western blot analysis after derivatization with DNPH [140]	(A) 1.22 ± 0.14 a.u.	(H) 0.60 ± 0.08 a.u.	(A) vs. (H) *p* < 0.05

Nguyen-Khoa et al. 2001 [132]	(A) 31 HD patients (mean age 64 ± 18 years, 15 M and 16 F) Dialysis vintage 6.0 ± 5.8 years	(H) 18 healthy subjects (age 45 ± 9 years, 8 M and 10 F)	Spectrophotometric assay after derivatization with DNPH [141]	(A) 0.55 ± 0.25 nmol/mg protein	(H) 0.37 ± 0.09 nmol/mg protein	(A) vs. (H) *p* < 0.01

Ward et al. 2003 [80]	22 HD patients divided into two groups depending on membrane composition (mean age 51 ± 5 years, 8 M and 4 F) Dialysis vintage 49 ± 11 months (A) Polysulfone membrane pre-HD (B) Polysulfone membrane post-HD (C) Cellulose triacetate membrane pre-HD (D) Cellulose triacetate membrane post-HD	(H) 17 healthy subjects (age range 23-54 years, both M and F)	ELISA after derivatization with DNPH (commercial kit)	(A) Pre-HD 0.144 ± 0.037 nmol/mg protein (B) Post-HD 0.175 ± 0.029 nmol/mg protein (C) Pre-HD 0.145 ± 0.030 nmol/mg protein (D) Post-HD 0.178 ± 0.035 nmol/mg protein	(H) 0.041 ± 0.008 nmol/mg protein	(A) vs. (H) *p* < 0.05 (C) vs. (H) *p* < 0.05

Danielski et al. 2003 [97]	36 HD patients divided into two groups: (A) 18 hypoalbuminemic patients (age 67.7 ± 13 years, 7 M and 11 F) (B) 18 normoalbuminemic patients (age 67.8 ± 11 years, 7 M and 1 F) Dialysis vintage unspecified	(H) 18 healthy subjects (age matched)	ELISA after derivatization with DNPH (commercial kit) Western blot analysis after derivatization with DNPH [140]	(A) Hypoalbuminemic patients 0.09 ± 0.02 nmol/mg protein (B) Normoalbuminemic patients 0.06 ± 0.01 nmol/mg protein	(H) 0.02 ± 0.01 nmol/mg protein	(A) vs. (H) *p* < 0.05 (B) vs. (H) *p* < 0.05

Pupim et al. 2004 [75]	(A) 50 HD patients (age 57.6 ± 17.2 years, 30 M and 20 F) Dialysis vintage unspecified	(H) 50 healthy subjects (age 49.7 ± 16.3 years, 18 M and 32 F)	ELISA after derivatization with DNPH (commercial kit)	(A) 0.154 ± 0.014 nmol/mg protein	(H) 0.029 ± 0.004 nmol/mg protein	(A) vs. (H) *p* < 0.001

Massy et al. 2003 [133]	(A) 22 HD patients (age 62 ± 19 years, 12 M and 10 F) Dialysis vintage 6.0 ± 5.8 years	(H) 12 healthy subjects (age 41 ± 8 years, 5 M and 7 F)	Spectrophotometric assay after derivatization with DNPH [141]	(A) 0.54 ± 0.17 nmol/mg protein	(H) 0.34 ± 0.09 nmol/mg protein	(A) vs. (H) *p* < 0.001

Köken et al. 2004 [77]	(A) 70 HD patients (age 49 ± 15 years, 33 M and 37 F) divided into six groups with different dialysis vintage: from 3-12 months (group 1) to 85–120 months (group 6)	(H) 12 healthy subjects (age 50 ± 5 years, 5 M and 7 F)	Spectrophotometric assay after derivatization with DNPH [141]	(A) 1.10 ± 0.20 nmol/mg protein The plasma PCO values in the six groups of ESRD patients on HD are shown in Figure 2 (no detailed values reported)	(H) 0.79 ± 0.01 nmol/mg protein	(A) vs. (H) *p* < 0.001

Anraku et al. 2004 [91]	22 HD patients (aged 25 to 87 years, 15 M and 7 F) Dialysis vintage 1-9 years (A) HD patients without intravenous iron administration (B) HD patients with intravenous iron administration	(H) 11 healthy subjects (age and gender matched)	Western blot analysis after derivatization with DNPH [140] Spectrophotometric assay after derivatization with fluoresceinamine [39]	No detailed values reported for WB analysis Spectrophotometric assay (A) 1.0 ± 0.1 nmol/mg protein (B) 2.2 ± 0.4 nmol/mg protein	(H) 0.40 ± 0.03 nmol/mg protein	(A) vs. (H) *p* < 0.05 (B) vs. (A) *p* < 0.05

Dursun et al. 2005 [81]	20 HD patients (age and sex unspecified) Dialysis vintage unspecified (A) Pre-HD (B) Post-HD	(H) 20 healthy subjects (age and sex unspecified)	Spectrophotometric assay after derivatization with DNPH [142]	(A) Pre-HD 0.889 ± 0.063 nmol/mg protein (B) Post-HD 0.997 ± 0.066 nmol/mg protein	(H) 0.417 ± 0.036 nmol/mg protein	(A) vs. (H) *p* < 0.05

Kalogerakis et al. 2005 [134]	(A) 22 HD patients (age 60.8 ± 17 years, 14 M and 8 F) Dialysis vintage unspecified	(H) 23 healthy subjects (age 42.5 ± 11.3 years, 12 M and 11 F)	ELISA after derivatization with DNPH [32]	(A) 0.15 ± 0.028 nmol/mg protein	(H) 0.093 ± 0.014 nmol/mg protein	(A) vs. (H) *p* < 0.01

Mera et al. 2005 [135]	(A) 20 HD patients (age 62.8 ± 12.7 years, 10 men and 10 women) Dialysis vintage 1-9 years	(H) 10 healthy subjects (67.8 ± 1.8 years, 6 M and 4 F)	Spectrophotometric assay after derivatization with fluoresceinamine [39]	(A) 3.12 ± 1.11 nmol/mg protein	(H) 2.10 ± 0.34 nmol/mg protein	(A) vs. (H) *p* < 0.01

Siems et al. 2005 [85]	107 HD patients divided into four groups with different Hb concentrations (A) Group 1: 13 patients with Hb < 8 g/dL (age 57 ± 12 years, 5 M and 8 F) (B) Group 2: 42 patients with Hb 8-10 g/dL (age 63 ± 14 years, 20 M and 22 F) (C) Group 3: 39 patients with Hb 10-12 g/dL (age 60 ± 15 years, 15 M and 19 F) (D) Group 4: 13 patients with Hb > 12 g/dL (age 61 ± 8 years, 6 M and 7 F)	(H) 80 healthy subjects (age 61 ± 14 years, 35 M and 45 F)	ELISA after derivatization with DNPH [143]	The plasma PCO values in the four groups of HD patients are shown in Figure 4 of Ref. [85] (nmol/mg protein) (no detailed values reported)	The plasma PCO values in the healthy control subjects are shown in Figure 4 of Ref. [85] (nmol/mg protein)	(A) vs. (H) *p* < 0.001 (B) vs. (H) *p* < 0.01 (C) vs. (H) *p* < 0.01 (D) vs. (H) *p* < 0.05

Lim et al. 2007 [90]	(A) 31 HD patients (age 57.2 ± 12.5 years, M) Dialysis vintage 4.6 ± 6.1 years	(H) 22 healthy subjects (age 53.4 ± 17.7 years, M)	Spectrophotometric assay after derivatization with DNPH [141]	(A) 10.5 ± 1.88 nmol/mg purified albumin	(H) 5.29 ± 1.21 nmol/mg purified albumin	(A) vs. (H) *p* < 0.001

Pieniazek et al. 2009 [82]	10 HD patients (mean age 58 ± 11 years, sex unspecified) Dialysis vintage unspecified (A) Pre-HD (B) Post-HD	(H) 9 healthy subjects (age 46 ± 15 years, sex unspecified)	Spectrophotometric assay after derivatization with DNPH [141]	(A) Pre-HD 2.27 ± 0.2 *μ*mol/L (B) Post-HD 2.94 ± 0.12 *μ*mol/L	(H) 0.67 ± 0.07 *μ*mol/L	(A) vs. (H) *p* < 0.0002 (B) vs. (H) *p* < 0.0002

Moradi et al. 2009 [124]	(A) 32 HD patients (mean age 51 ± 2.5 years, 22 M and 10 F) Dialysis vintage unspecified	(H) 13 healthy subjects (age-matched, 9 M and 4 F)	Spectrophotometric assay after derivatization with DNPH (commercial kit)	The plasma PCO values in HD patients are shown in Figure 1 (no detailed values reported) (A) ~11.00 ± 4.00 nmol/mg protein	The plasma PCO values in healthy control subjects are shown in Figure 1 (no detailed values reported) (H) ~0.50 ± 1.00 nmol/mg protein	(A) vs. (H) *p* < 0.05

Koca et al. 2010 [78]	111 HD patients divided into four groups according to HD duration: (A) Group 1: (*n* = 31, age 53 ± 14 y, 14 M and 17 F) Dialysis vintage 0-2 years (B) Group 2: (*n* = 40, age 55 ± 17 y, 19 M and 21 F) Dialysis vintage 3-5 years (C) Group 3: (*n* = 27, age 56 ± 14 y, 12 M and 15 F) Dialysis vintage 6-8 years (D) Group 4: (*n* = 13, age 47 ± 9 y, 6 M and 7 F) Dialysis vintage 9-11 years	(H) 24 healthy subjects (age 48 ± 10 years, 10 M and 14 F)	Spectrophotometric assay after derivatization with DNPH [141]	The plasma PCO values in HD patients are shown in Figure 2 (no detailed values reported) (*μ*mol/L)	The plasma PCO values in healthy subjects are shown in Figure 2 (no detailed values reported) (*μ*mol/L)	No significant variations

Terawaki et al. 2010 [86]	83 anuric HD patients divided into with or without CVD: (A) Patients with CVD pre-HD (B) Patients with CVD post-HD (*n* = 66), age 63.5 ± 12.5 years, 32 M and 34 F Dialysis vintage 85.0 ± 64.6 months (C) Patients without CVD pre-HD (D) Patients without CVD post-HD (*n* = 20), age 74.3 ± 12.8 years, 11 M and 9 F Dialysis vintage 58.3 ± 33.3 months	—	Spectrophotometric assay after derivatization with DNPH (commercial kit)	Patients with CVD (A) Pre-HD 0.81 ± 0.16 nmol/mg protein (B) Post-HD 0.53 ± 0.13 nmol/mg protein patients without CVD (C) Pre-HD 0.82 ± 0.17 nmol/mg protein (D) Post-HD 0.58 ± 0.16 nmol/mg protein	—	No significant variations

Pavone et al. 2011 [37]	14 HD patients (age 72 ± 10 years, 7 M and 7 F) Dialysis vintage 50 ± 25 months (A) Pre-HD (B) Post-HD	—	Western blot analysis after derivatization with DNPH Carbonylated protein identification was carried out by MALDI-TOF/MS mass fingerprinting	The plasma PCO values before and after HD are shown in Figure 2 (no detailed values reported) (A) ~5900 ± 1200 a.u. (B) ~7100 ± 2000 a.u.	—	

Albarello et al. 2012 [83]	23 HD patients (9 men and 14 women, mean age 50.8 ± 17.3 years) Dialysis vintage unspecified (A) Pre-HD (B) Post-HD	—	Spectrophotometric assay after derivatization with DNPH [141]	(A) Pre-HD 0.62 ± 0.14 nmol/mg protein (B) Post-HD 0.86 ± 0.16 nmol/mg protein	—	(A) vs. (B) *p* < 0.001

Almeida et al. 2013 [137]	(A) 35 HD patients (18 years old or older, 16 M and 19 F) Dialysis 26.0 (35.0-13.0) month (a)	(H) 35 healthy subjects paired to age and gender	Spectrophotometric assay after derivatization with DNPH [141]	(A) 1.9 (2.6-1.3) nmol/mg protein	(H) 0.9 (1.5-0.7) nmol/mg protein	(A) vs. (H) *p* < 0.001

Caimi et al. 2013 [47]	(A) 31 HD patients (61.5 ± 12.8 years, 16 men and 15 women) Dialysis vintage 48.5 ± 35.7 months	(H) 26 healthy subjects (age 43.54 ± 6.92 years, 17 M and 9 F)	ELISA after derivatization with DNPH (commercial kit)	(A) 1.230 ± 0.192 nmol/mg protein	(H) 0.440 ± 0.134 nmol/mg protein	(A) vs. (H) *p* < 0.001

Murillo-Ortiz et al. 2016 [65]	(A) 35 HD patients with ferritin levels < 500 ng/mL (age 45.4 ± 16.6 years, 17 M and 18 F) Dialysis vintage 40.87 ± 41.65 months (A) 35 HD patients with ferritin levels > 500 ng/mL (age 46.5 ± 16.9 years, 17 M and 18 F) Dialysis vintage 20.17 ± 29.00 months	—	Spectrophotometric assay after derivatization with DNPH [141]	(A) HD patients with ferritin < 500 ng/mL 22.5 ± 5.4 ng/*μ*L (B) HD patients with ferritin > 500 ng/mL 27.2 ± 5.2 ng/*μ*L	—	(A) vs. (B) *p* < 0.0004

Colombo et al. 2018 [41]	69 HD patients (mean 69 ± 1.5 years, 42 M and 24 F) Dialysis vintage 5.8 ± 0.46 years (A) M pre-HD (B) M post-HD (C) F pre-HD (D) F post-HD	—	ELISA after derivatization with DNPH (commercial kit)	(A) M pre-HD 0.118 ± 0.016 nmol/mg protein (B) M post-HD 0.118 ± 0.013 nmol/mg protein (C) F pre-HD 0.1348 ± 0.0267 nmol/mg protein (D) F post-HD 0.1604 ± 0.0313 nmol/mg protein	—	(C) vs. (D) *p* < 0.01

Data are presented as the mean ± standard deviation (SD), in the reported studies. Dialysis vintage (length of time on dialysis) is presented as months or years. M: male; F: female; CVD: cardiovascular disease; CKD: chronic kidney disease; ESRD: end-stage renal disease; HD: haemodialysis (haemodialysed); PCO: protein carbonyls (carbonylated proteins); DNPH: 2,4-dinitrophenylhydrazine; a.u.: arbitrary units.

**Table 3:** Comparison of plasma PCO levels in PD and HD.

Study year	Number of HD or PD patients (age and sex) and dialysis vintage	Number of control healthy subjects (age and sex)	Analytical methods	Plasma PCO in HD or PD patients	Plasma PCO in control subjects	*p* value
Tbahriti et al. 2013 [50]	(A) 40 HD patients (age 42 ± 11 years, 22 M and 18 F) (B) 25 PD patients (age 39 ± 15 years)	—	Spectrophotometric assay after derivatization with DNPH (commercial kit)	(A) HD patients 1.85 ± 0.16 nmol/mg albumin (B) HD patients 1.92 ± 0.13 nmol/mg albumin	—	Statistical difference not specified

Erdoğan et al. 2002 [108]	(A) 7 HD patients (B) 9 PD patients (mean age 38.7 ± 12.9 years, 7 M and 9 F)	(H) 9 age-matched healthy subjects (2 M and 7 F)	Spectrophotometric assay after derivatization with DNPH [142]	(A) HD patients 1.1 ± 0.2 nmol/mg protein (B) PD patients 1.1 ± 0.3 nmol/mg protein	(H) 0.8 ± 0.3 nmol/mg protein	No significant variations

Doñate et al. 2002 [109]	(A) 21 HD patients (B) 42 PD patients	—	ELISA after derivatization with DNPH [32]	(A) HD patients 0.1665 ± 0.04 nmol/mg protein (B) PD patients 0.1452 ± 0.03 nmol/mg protein	—	(A) vs. (B) *p* < 0.004

Mitrogianni et al. 2009 [44]	(A) 25 HD patients (age 26–80 years, 16 M and 9 F) (B) 21 PD patients (age 18–77 years, 13 M and 8 F)	(H) 20 healthy subjects (age range 29–78 years, 12 M and 8 F)	Western blot analysis after derivatization with DNPH (commercial kit)	(A) HD patients densitometric units (B) PD patients densitometric units	Control densitometric units Control densitometric units	(A) vs. (H) *p* < 0.05

Mekki et al. 2010 [110]	(A) 20 HD patients (age 36 ± 12 years, 8 M and 12 F) HD vintage 12-60 months (B) 20 PD patients (age 40 ± 8 years, 10 M and 10 F) PD vintage 3-48 months	—	Spectrophotometric assay after derivatization with DNPH [141]	(A) HD patients 0.92 ± 0.15 *μ*mol∗mL (B) PD patients 1.90 ± 0.10 *μ*mol∗mL	—	(A) vs. (B) *p* < 0.01

Guo et al. 2011 [111]	(A) 45 PD patients (age 54 ± 9 years, 22 M and 23 F) PD vintage 2.4 ± 1.1 years	(H) 30 healthy subjects (age 52 ± 7 years, 12 M and 18 F)	Spectrophotometric assay after derivatization with DNPH [141]	(A) PD patients 0.42 ± 0.23 nmol/mg protein	(H) 0.16 ± 0.08 nmol/mg protein	(A) vs. (H) *p* < 0.05

Tbahriti et al. 2013 [50]	(A) 40 HD patients (age 42 ± 11 years, 22 M and 18 F) HD vintage 14-109 months (B) 25 PD patients (age 39 ± 15 years, 12 M and 13 F) PD vintage 5-49 months	—	Spectrophotometric assay after derivatization with DNPH (commercial kit)	(A) HD patients 1.85 ± 0.16 nmol/mg protein (B) PD patients 1.92 ± 0.13 nmol/mg protein	—	

Data are presented as the mean ± standard deviation (SD), in the reported studies. M: male: F: female; CKD: chronic kidney disease; ESRD: end-stage renal disease; HD: haemodialysis (haemodialysed); PCO: protein carbonyls (carbonylated proteins); DNPH: 2,4-dinitrophenylhydrazine; PD: peritoneal dialysis.

## Abbreviations

BUN: Blood urea nitrogen
CKD: Chronic kidney disease
CRP: C-reactive protein
CT: Conservative therapy
CVD: Cardiovascular disease
DNP: 2,4-Dinitrophenylhydrazone
DNPH: 2,4-Dinitrophenylhydrazine
ELISA: Enzyme-linked immunosorbent assay
ESRD: End-stage renal disease
GFR: Glomerular filtration rate
HD: Haemodialysis
HRP: Horseradish peroxidase
KDIGO: Kidney Disease Improving Global Outcomes
KT: Kidney transplantation
MALDI-TOF: Matrix-assisted laser desorption/ionization time-of-flight
MS: Mass spectrometry
PCO: Protein carbonyls (carbonylated proteins)
PD: Peritoneal dialysis
PVDF: Polyvinylidene fluoride membrane
ROS: Reactive oxygen species
RRF: Residual renal function
RRT: Renal replacement therapy.

## Supplementary Materials

Supplementary Materials Table 4: patient selection. CKD or RRT inclusion and/or exclusion criteria in various studies [37, 41, 45–52, 66, 76, 78, 79, 81–84, 86, 87, 90–92, 98, 109–112, 116, 132–139].
