# Supplementary material for: Plasma Protein Carbonyls as Biomarkers of Oxidative Stress in Chronic Kidney Disease, Dialysis, and Transplantation
Source: Oxid Med Cell Longev. 2020 Nov 24;2020:2975256. doi: 10.1155/2020/2975256 (PMC7707964; doi:10.1155/2020/2975256)
Supplement: Supplementary Materials — Table 4: patient selection. CKD or RRT inclusion and/or exclusion criteria in various studies [37, 41, 45–52, 66, 76, 78, 79, 81–84, 86, 87, 90–92, 98, 109–112, 116, 132–139]. [file 2975256.f1.docx]

| **Study year** | **Aim of the study** | **CKD patients selection (inclusion and/or exclusion criteria)** |
| --- | --- | --- |
| **Himmelfarb et al. 2000**  **[132]** | To evaluate plasma PCO levels in HD or PD patients and CKD patients not receiving RRT, compared to healthy subjects. | Inclusion criteria were CKD and RRT achieved by HD or PD.  Other inclusion and/or exclusion criteria were unspecified. |
| **Himmelfarb and McMonagle 2001**  **[90]** | To evaluate plasma PCO levels in HD patients and CKD patients not receiving RRT. | Inclusion criteria were CRF and RRT achieved by HD.  Other inclusion and/or exclusion criteria were unspecified. |
| **Nguyen-Khoa et al. 2001**  **[133]** | To evaluate plasma PCO levels in HD patients compared to healthy subjects | Exclusion criteria were smoke, patients with chronic hepatitis, haematological and inflammatory disorders, cancer or immunosuppressive therapy. |
| **Erdogan et al. 2002**  **[109]** | To evaluate plasma PCO levels in HD or PD patients compared to healthy subjects | Inclusion criterion was RRT achieved by HD or PD.  Other inclusion and/or exclusion criteria were unspecified. |
| **Doñate et al. 2002 [110]** | To evaluate plasma PCO levels in HD or PD patients compared to healthy subjects | Inclusion criterion was RRT achieved by HD or PD.  Other inclusion and/or exclusion criteria were unspecified. |
| **Ward et al. 2003 [81]** | To evaluate plasma PCO levels before and after HD. | Inclusion criteria were a stable HD prescription, a well-functioning native fistula or  Gore-Tex graft and an absence of clinical evidence of infection during the month prior to the study. |
| **Massy et al. 2003 [134]** | To evaluate plasma PCO levels in HD patients compared to healthy subjects | Exclusion criteria were smoke, chronic hepatitis, haematological and inflammatory disorders, cancer or immunosuppressive therapy. |
| **Danielski et al. 2003**  **[98]** | To evaluate plasma PCO levels in hypoalbuminemic and normoalbuminemic HD patients compared to healthy subjects | Inclusion criteria were serum albumin levels of 3.2 g/dL or less without a precipitating cause, such as recent hospitalization or known infection. Exclusion criteria consisted of inability to give informed consent, overt infections, or known metastatic cancer. |
| **Pupim et al. 2004 [76]** | To examine how initiation of maintenance HD affects plasma PCO levels | There were no exclusion criteria (except age <18 years),. |
| **Oberg et al. 2004**  **[46]** | To evaluate plasma PCO levels in CKD patients on stages 3 to 5 compared to healthy subjects | Inclusion criteria were CKD stage 3-5.  Other inclusion and/or exclusion criteria were unspecified. |
| **Köken et al. 2004 [78]** | To examine the effect of prolonged HD treatment on plasma PCO levels | Exclusion criteria were smoke, evidence of rapidly progressive renal failure or partial recovery of renal function, overt malnutrition, diabetes mellitus, acute or chronic superimposed inflammatory disorders, erythropoietin-resistant anaemia and the use of any anti-oxidant vitamin or fish-oil supplement within the last three months. |
| **Anraku et al. 2004 [92]** | To examine the effect of intravenous iron administration on plasma PCO levels in HD patients | All patients were not treated with antioxidants such as vitamin E and C in the three months before inclusion onto the study. |
| **Dursun et al. 2005 [82]** | To evaluate plasma PCO levels before and after HD in ESRD patients compared to healthy subjects | Inclusion criterion was RRT achieved by HD.  Other inclusion and/or exclusion criteria were unspecified. |
| **Simmons et al. 2005**  **[116]** | To evaluate plasma PCO levels before and after kidney transplantation | Exclusion criteria were acute illnesses or a previous renal transplant. |
| **Kalogerakis et al. 2005**  **[135]** | To evaluate plasma PCO levels in HD patients compared to healthy subjects | Inclusion criterion was RRT achieved by HD.  Other inclusion and/or exclusion criteria were unspecified. |
| **Mera et al. 2005 [136]** | To evaluate plasma PCO levels in HD patients compared to healthy subjects | RRT achieved by HD was the only inclusion criterion. |
| **Siems et al. 2005 [86]** | To evaluate plasma PCO levels before and after HD. PCO levels were related to the degree of renal anaemia in HD patients. | Exclusion criteria were smoke, supplementation of antioxidant substances, acute infections and acute phases of rheumatological diseases. |
| **Lim et al.2007**  **[91]** | To evaluate plasma PCO levels in HD patients compared to healthy control subjects | Exclusion criteria were malignancy, collagen vascular diseases and acute infectious or inflammatory illnesses. |
| **Puchades Montesa et al. 2009**  **[47]** | To evaluate plasma PCO levels in CKD patients on stage 4 compared to healthy subjects | Inclusion criterion was patients who had been clinically stable during the previous six months. Exclusion criteria were neoplastic condition, copious bleeding, inflammatory illness or an active infection, and treatment with intravenous iron in the three preceding months. |
| **Pieniazek et al 2009**  **[83]** | To evaluate plasma PCO levels before and after HD | Inclusion criterion was RRT achieved by HD.  Other inclusion and/or exclusion criteria were unspecified. |
| **Matsuyama et al. 2009**  **[49]** | To evaluate plasma PCO levels in CKD patients on stage 1 to 5. | Inclusion criteria were CKD stage 1-5.  Other inclusion and/or exclusion criteria were unspecified. |
| **Mitrogianni et al. 2009**  **[45]** | To evaluate carbonylated albumin levels in CKD patients on stage 2 to 4 and in HD or PD patients compared to healthy subjects | Exclusion criteria were acute or chronic inflammation caused by any virulent agent, vitamin supplementation, and smoking. |
| **Moradi et al. 2009 [137]** | To evaluate plasma PCO levels in HD patients compared to healthy subjects | Exclusion criteria were evidence of acute or chronic infection or acute intercurrent illnesses. |
| **Mekki et al. 2010 [111]** | To investigate the effects of HD and PD on plasma PCO levels. | Exclusion criteria were systemic disease such as diabetes, liver disorders and patients affected by nephrotic syndrome. None of the patients was taking lipid-lowering drugs or antioxidant supplements. |
| **Aveles et al. 2010 [50]** | To evaluate plasma PCO levels in patients with CKD stage 2 to5, and before and after kidney transplantation. | Exclusion criteria were clinical signs of active infection, liver dysfunction, autoimmune diseases, malignancy, and the use of anti-inflammatory or immunosuppressant drugs three months prior to recruitment. |
| **Koca et al. 2010 [79]** | To evaluate plasma PCO levels in HD patients with different HD duration compared to healthy subjects | Exclusion criteria were evidence of rapidly progressive renal failure or partial recovery of renal function, overt malnutrition, acute or chronic superimposed inflammatory disorders including urinary tract infection and viral hepatitis, hemolytic disorders, rhabdomyolysis, amyloidosis, acute cardiovascular problems, hepatic or respiratory disease, erythropoietin-resistant anemia, and the use of any antioxidant vitamin or fish-oil supplement within the previous three months. |
| **Terawaki et al. 2010**  **[87]** | To evaluate plasma PCO levels before and after HD. | Exclusion criteria were history of apparent myocardial infarction or stroke, apparent infection, bleeding, liver dysfunction, collagen disease, systemic vasculitis, or malignancies. |
| **Pavone et al. 2011 [37]** | To examine the targets of plasma protein carbonylation in HD patients. | Exclusion criteria were diabetes, acute infection or blood transfusion in the past three months, smoking, unsteady erythropoietin dosage, and a history of malignancy. |
| **Guo et al. 2011 [112]** | To assess the relationship between copper/zinc ratio and PCO levels in the plasma of PD patients. | Exclusion criteria were liver disease, mental retardation, dementia, psychiatric illness, or cancer, and administration of immune suppressant drugs or supplementation with antioxidant vitamins/minerals (e.g. selenium, Cu, and Zn). All patients were clinically stable and free of edema. |
| **Albarello et al. 2012**  **[84]** | To evaluate plasma PCO levels before and after HD. | Exclusion criteria were malignant disease, infectious disease, and liver disease. |
| **Boudouris et al. 2013**  **[139]** | To evaluate plasma PCO levels in PD patients and the effect of treatment with ascorbic acid and alpha-tocopherol. | Exclusion criteria were incidence of peritonitis or any other infection, exacerbation of any inflammatory disease at least four weeks preceding blood sampling, presence of any chronic inflammatory disease, use of non-steroidal anti-inflammatory drugs, allopurinol or antioxidants, insufficient regulation of diabetes, if in the initial measurement variations occur as for the parameters studied. |
| **Almeida et al. 2013**  **[138]** | To evaluate plasma PCO levels in HD patients compared to healthy subjects | Inclusion criteria were 18 years old or older, six or more months on dialysis treatment, absence of malignancy, acute inflammatory diseases, vasculitis, and haemoglobinopathies. |
| **Tbahriti et al. 2013**  **[51]** | To evaluate PCO levels in HD or PD patients compared to patients with CKD stage 1-4 | Exclusion criteria included clinical signs of infection (hepatitis B and hepatitis C), malignancy, active immunological diseases, and immunosuppressive or immunomodulatory and anti-inflammatory drugs administration, patients with diabetes and nephrotic syndrome. |
| **Caimi et al. 2013 [48]** | To evaluate PCO levels in CKD patients on conservative therapy and HD patients compared to healthy subjects | Inclusion criteria were conservative theraphy or replacement therapy achieved by HD.  Other inclusion and/or exclusion criteria were unspecified. |
| **Murillo-Ortiz et al. 2016**  **[66]** | To evaluate impact of oxidative stress in premature ageing and iron overload in HD patients | Exclusion criteria were immune disorders and the habit of smoking and alcoholism. |
| **Drożdż et al. 2016 [52]** | To evaluate PCO levels in patients with CKD stage 1-4 | Exclusion criteria were lack of consent of the patient or parents, congenital heart defects or other primary heart diseases, acute infections, or failure of other organs. |
| **Colombo et al. 2018**  **[41]** | To evaluate plasma PCO levels before and after HD in men and women. | The presence of a clinically overt infectious process was the only exclusion criterion. |

**Supplementary Material**

Table 4: Patient selection. CDK or RRT inclusion and/or exclusion criteria in various studies.
